# The short-term effects of hydrotherapy on pain and self-perceived functional status in individuals living with osteoarthritis of the knee joint

**DOI:** 10.4102/sajp.v75i1.476

**Published:** 2019-07-24

**Authors:** Kganetso Sekome, Stacey Maddocks

**Affiliations:** 1 School of Therapeutic Sciences, Department of Physiotherapy, University of the Witwatersrand, Johannesburg, South Africa; 2 School of Health Sciences, Department of Physiotherapy, University of KwaZulu-Natal, Durban, South Africa

**Keywords:** knee osteoarthritis, pain, functional status, hydrotherapy, exercise

## Abstract

**Background:**

People living with knee osteoarthritis (OA) commonly present with difficulty in walking long distances, ascending and descending stairs or rising from sitting. These functional limitations have been shown to have a negative effect on their overall activities of daily living.

**Objectives:**

The aim of this study was to determine the effects of a 4-week hydrotherapy programme on measures of pain and self-perceived functional status in individuals living with knee OA.

**Method:**

A total of 18 participants with chronic knee OA participated in this study. Participants completed 4 weeks of hydrotherapeutic intervention provided by an independent physiotherapist. Outcome measures for the study included pain assessed using the visual analogue scale (VAS) and self-perceived functional status using the Western Ontario and McMaster Universities Osteoarthritis Index (WOMAC). Outcome measures were assessed at baseline and after the 4 weeks of intervention.

**Results:**

The 4-week hydrotherapy programme resulted in a significant decrease in pain and a significant improvement in self-perceived functional status in all participants. There was a statistically significant mean decrease in VAS scores of 3.72 (± 2.45), *p* ≤ 0.05, with a 95% confidence interval ranging from 2.506 to 4.938. There was also a statistically significant mean decrease in WOMAC scores of 29.5 (± 15.51), *p* ≤ 0.05. with a 95% confidence interval ranging from 21.788 to 37.212.

**Conclusion:**

This study demonstrated that a 4-week hydrotherapeutic exercise programme results in significantly reduced pain and improved self-perceived functional status in individuals living with knee OA.

**Clinical implications:**

Four weeks of hydrotherapy exercises twice a week in a heated pool can significantly decrease pain and improve functional status in individuals with knee OA.

## Introduction

People living with knee osteoarthritis (OA) commonly present with difficulty in walking long distances, ascending and descending stairs or rising from sitting (Cross et al. 2016). These functional limitations have been shown to have a negative effect on their overall activities of daily living (ADL) (Blagojevic et al. 2010).

With the increasing prevalence of obesity worldwide and particularly in the Southern African region (Ahima 2016) and the adoption of more sedentary lifestyles in recent years, the prevalence of OA has been estimated to have increased significantly (Hodkinson & Mohammed 2011). Knee OA is the most common occurring arthritis of all joints, with a prevalence ranging from 67% to 70% and an early onset (≥ 20 years) based on a worldwide systematic review (Pereira et al. 2011). Osteoarthritis primarily affects the large weight-bearing joints such as the knee and hip, resulting in pain, loss of movement and loss of normal functioning (Kenyon & Kenyon 2009). Osteoarthritis may be characterised by joint pain, joint stiffness, joint instability, crepitus on movement, bony swellings, decreased range of motion, muscle weakness and loss of physical function (Manheim et al. 2012; Walker 2011). These characteristics of OA may result from the restriction in joint movement due to joint capsular thickening and the presence of osteophytes (Walker 2011).

Current therapies in the treatment of OA are largely directed towards pain management and reduction of functional limitations (Ickinger & Mohammed 2010). The aim of knee OA management is to improve the quality of life, slow down the progression of the disease, and improve and maintain the functioning of the patient by managing pain, stiffness and other associated symptoms. Modern guidelines recommend non-pharmacological interventions such as physical activity exercises and self-management interventions like weight control, as first-line options in the management of OA. Currently, physical activity exercises for OA may be provided on land or in an aquatic environment, such as hydrotherapy (Walker 2011). Aquatic or hydrotherapeutic exercises arguably offer more benefits than land-based physical activity exercises in decreasing joint overload, decreasing chances of injury and significantly decreasing pain experienced by individuals with OA (Vaile et al. 2008).

Hydrotherapy has been shown to have positive outcomes on multiple body system functions, including cardiovascular, pulmonary, metabolic and musculoskeletal functioning (Mooventhan & Nivethitha 2014).

Most studies published on the effectiveness of hospital-based hydrotherapy treatment focused on the long-term effects of the intervention, and there is little evidence published on the short-term effects of hospital-based hydrotherapy treatment (Dias et al. 2017; Lin, Davey & Cochrane 2004; Silva et al. 2008). The aim of this study was to determine the effects of a 4-week hydrotherapy programme on pain and self-perceived functional status in individuals living with OA of the knee joint.

## Methods

This study utilised a quantitative pre-test and post-test design. The study was conducted at a tertiary-level public hospital in KwaZulu-Natal province, South Africa. A convenience sample of 18 participants from an initial cohort of 36 participants diagnosed with knee OA completed both the pre-test and post-test assessment.

Screening of the participants was carried out by one of the authors and research assistants (M.C., L.K., P.G., L.P.) to determine inclusion in the study. Men and women aged 37 years and older with radiological evidence of knee OA, experiencing knee pain for most days of the preceding month, crepitus on active joint motion, morning stiffness for less than 30 min in duration and bony enlargements of the knee on examination were included in the study. Participants had to experience knee pain ranging from 3 to 10 on average on VAS and have been willing to comply with the follow-up assessment and treatment. Participants who presented with hydrophobia, skin or other connective tissue diseases affecting the knee; severe systemic disease that could interfere with the assessment; epilepsy, serious neurologic diseases or psychiatric symptoms; and red, hot, swollen joints during screening were excluded from the study. Pregnant females were excluded from the study because of the water temperature and the intensity of the hydrotherapy exercises (Hinman, Heywood & Day 2007). Participants who were unable to walk and those attending less than 50% of the intervention were also excluded from the data analysis.

### Ethical considerations

Ethical clearance to conduct this study was obtained from the Biomedical Research Ethics Committee (BREC) of the University of KwaZulu-Natal (ethical clearance number: SHSEC 031/12). Permission from the Department of Health province of KwaZulu-Natal and the Acting Director at the study site was obtained. Written informed consent was obtained from all participants prior to participation in the study.

### Outcome measurement tools

The primary outcome measure was pain, which was assessed using a 10-cm visual analogue scale (VAS). This is a valid and reliable (*r* = 0.98), and responsive technique for assessing perceived pain in participants with OA (Cheing, Hui-Chan & Chan 2002; Schencking et al. 2009).

The secondary outcome measure was self-perceived functional status, and this was assessed using the Western Ontario and McMaster Universities Osteoarthritis Index (WOMAC), which consists of three subscales: pain, stiffness and physical function. This is a well-recognised valid, reliable and responsive measurement tool (Cronbach’s coefficient alpha of 0.91, 0.81 and 0.84) (Giaquinto et al. 2010; Silva et al. 2008; Yildirim, Filiz Ulusoy & Bodur 2010). The WOMAC test–retest reliability was satisfactory with ICCs of 0.86, 0.68 and 0.89, respectively (Salaffi et al. 2003). All outcome measures were assessed at baseline and at 4 weeks after the hydrotherapy intervention was completed.

### Intervention

The hydrotherapy programme was based on a protocol by Hinman et al. (2007), which is composed of functional weight-bearing and progressive exercises that were provided twice a week for 60 minutes. Warm-up and cool-down exercises were included in the programme. A physiotherapist employed full-time at the study site with experience in hydrotherapy and not involved in data collection instructed participants in the hydrotherapy pool.

The physiotherapist was trained in first aid and could respond to any adverse events that may have resulted from the hydrotherapy exercise. No adverse events were observed or reported during the intervention. The water temperature was set at 34°C (Hinman et al. 2007). Quality of movement was emphasised. Feedback was provided by the physiotherapist to the participants regarding posture and emphasis was placed on controlling the upper body when standing or during movement. Patients’ attendance at each session was recorded. The hospital’s hydrotherapy safety protocol was followed during each intervention.

During the period of the study, participants were encouraged to continue with their lives as normal, to not undergo any additional hydrotherapy exercises and to consult with the physiotherapist who administered the hydrotherapy intervention if they experienced any increased pain following a hydrotherapy session. Upon completion of the 4-week programme, participants were encouraged to continue hydrotherapy exercises twice a week at the study site with the physiotherapist who administered the hydrotherapy intervention during data collection. This was done because of the reported reduction in pain and improved physical function during the intervention. The physiotherapist was encouraged to provide home exercise programmes to the patients where necessary to increase self-efficacy (Table 1).

**TABLE 1:** Hydrotherapy exercise programme.

Session (day)	Water depth	Lower limb exercises	Sets and repetitions (each leg)	Walking (min)
1	ASIS	(1) Double-leg squats	2 × 10	6
		(2) Double-leg calf raises	2 × 10	
		(3) Dynamic lunge	2 × 10	
2	ASIS	As for session 1	As for session 1	8
3	ASIS	As for session 1, plus:		10
		(4) Single-leg stance, contralateral knee flexion followed by extension	2 × 10	
		(5) Single-leg stance, contralateral hip abduction followed by adduction	2 × 10	
		(6) Single-leg stance, contralateral hip hitching	2 × 10	
4	ASIS	(1) Single-leg squats	2 × 10	10
		(2) Single-leg calf raises	2 × 10	
		(3) Dynamic lunge Plus exercises 4, 5 and 6 from session 3.	2 × 10	
5	ASIS	As for session 4, plus: (7) Step-ups	2 × 10	10
6	ASIS	As for session 5, but modify: (8) Step downs	2 × 10	10
7	ASIS	As for session 6, but for exercises 4 and 5, increase the speed (resistance) of moving leg as able	2 × 10 followed by 1 × 5	10
8	ASIS	As for session 7	3 × 10	10

*Source*: Hinman, R.S., Heywood, S.E. & Day, A.R., 2007, ‘Aquatic physical therapy for hip and knee osteoarthritis: Results of a single-blind randomized controlled trial’, *Physical Therapy* 87(1), 32–43. https://doi.org/10.2522/ptj.20060006

ASIS, anterior superior iliac spine.

### Data analysis

Data from the study were captured and analysed using the Statistical Package for Social Sciences (SPSS version 19). The alpha level was set at *p* = 0.05, while 95% confidence intervals were calculated. Descriptive statistics were used to describe the data and to obtain the mean and standard deviations (SD). Cronbach’s alpha reliability analysis was conducted to determine the internal consistency or reliability of the WOMAC subscales. The Cronbach’s alpha for the WOMAC subscales were pain: 0.874, stiffness: 0.754 and ADL 0.944 (Table 2).

**TABLE 2:** Cronbach’s alpha scores pre- and post-intervention for Western Ontario and McMaster Universities Osteoarthritis Index.

Variables	Cronbach’s alpha	Cronbach’s alpha based on standardised items	No. of items
Pre-pain	0.807	0.808	5
Pre-stiffness	0.235	0.235	2
Pre-ADL	0.949	0.947	16
Post-pain	0.874	0.885	5
Post-stiffness	0.754	0.759	2
Post-ADL	0.944	0.946	16

ADL, activities of daily living.

STATA version 15.1 was used to test for skewness of data in terms of participants’ age.

## Results

**FIGURE 1:**
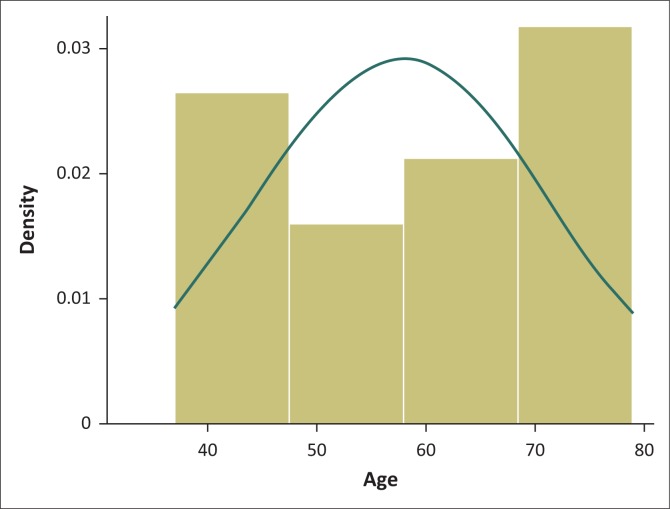
Normality histogram testing skewness of participants’ age.

A total of 36 possible participants with diagnosed knee OA were screened. Of these, 15 were excluded from the study as they did not meet the inclusion criteria. Six patients were post-total knee replacement, five patients presented with a combination of hip and knee OA, two presented with hydrophobia, one patient was not able to attend because of being employed and one had previously been diagnosed with epilepsy. The remaining 21 volunteers ranging 37 to 79 years old participated in the intervention. During the 4 weeks of the intervention, all but three participants (14.28%) attended 100% of the hydrotherapy classes; these three had an attendance of less than 50% because of transport difficulties and social responsibilities. Eighteen (85.71%) participants were included in the final results and post-test assessment. Sixteen (88.89%) participants were female and two (11.11%) were male. The mean age of study participants was 57.7 (± 13.6) years. Figure 1 is a normality histogram; the histogram shows that the age of the participants is not skewed and is normal.

A paired samples *t* test was conducted to evaluate the impact of the intervention on reported pain (VAS) and self-perceived functional status (WOMAC) of the participants. Table 3 shows that there was a statistically significant mean decrease in VAS scores of 3.72 (± 2.45), *p* ≤ 0.05, with a 95% confidence interval ranging from 2.506 to 4.938. The effect size = 0.71 was large (Fritz, Morris & Richler 2012). There was also a statistically significant mean decrease in WOMAC scores of 29.5 (± 15.51). Effect size = 0.79 was large (Fritz et al. 2012). The mean differences and standard error of the mean (SEM) of the three reported subscales of WOMAC (pain, stiffness and ADL) are also presented in Table 3.

**TABLE 3:** Mean differences of the pre- and post-test in Visual Analogue Scale and Western Ontario and McMaster Universities Osteoarthritis Index scores.

Variable	Mean differences						
	Mean	SD	SEM	95% confidence interval of the difference		*p*	Effect size
				Lower	Upper		
VAS pre-intervention	3.722	2.445	0.576	2.506	4.938	0.042	0.71
VAS_post-intervention							
WOMAC_pre-intervention	29.500	15.508	3.655	21.788	37.212	0.591	0.79
WOMAC_post-intervention							
Subscales of WOMAC							
Pain pre-intervention	6.333	3.742	0.882	4.473	8.194	0.159	0.75
Pain post-intervention							
Stiff pre-intervention	2.667	1.372	0.323	1.984	3.349	0.093	0.8
Stiff post-intervention							
ADL_pre-intervention	20.500	11.597	2.734	14.733	26.267	0.052	0.77
ADL_post-intervention							

SD, standard deviation; SEM, standard error of the mean; WOMAC, Western Ontario and McMaster Universities Osteoarthritis Index.

## Discussion

This study was designed to examine the effects of a 4-week hydrotherapy programme on measures of pain and self-perceived functional status in patients with OA of the knee joint. There has been little research done to examine the short-term effects of hydrotherapy on pain and self-perceived functional status in individuals with knee OA (Dias et al. 2017; Lin et al. 2004; Silva et al. 2008). The results of this study show that a twice-weekly hydrotherapy programme for 4 weeks can produce a statistically significant decrease in pain, stiffness and an improvement in functional ability in individuals who comply with the programme. These results are contrary to the results of a longer single-blinded randomised controlled trial conducted among geriatrics with knee OA, which aimed to determine the efficacy of land versus aquatic exercises on pain and function (Lund et al. 2008). The younger age of our study participants (Prestmo et al. 2015) could be a potential reason for the difference in findings. Lund et al. (2008) found that only land-based exercise demonstrated some improvement in pain and muscle strength compared with the aquatic exercise group, no clinical benefits were evident immediately after the aquatic exercise (8 weeks). At 3 months of follow-up, there was a reduction of pain in the land-based exercise group but no improvement in the hydrotherapy group. Our study did not have a control group and only took measurements before and immediately after the intervention and this is a limitation of our study. Results may have been more comparable if the participants were followed up and outcome measures taken again at 3 months after the intervention. Hinman et al. (2007) recommended that follow-up studies targeted at evaluating the characteristics of people who respond to land- and water-based exercises is necessary to determine whether certain subgroups of people benefit more from the one or the other regime.

This study had more female participants than male, and this could have influenced the results (Dias et al. 2017). Women in the South African population live a healthier and more active lifestyle compared with males especially when made aware of their health status (Mayosi et al. 2009; Micklesfield et al. 2014). The lifestyle behaviour patterns for participants in this study were not determined and should be a consideration for future similar studies.

The prevalence of knee OA is significantly higher in females than males (Bartley et al. 2016; Dias et al. 2017), and thus interventions that will decrease the burden on the affected population need to be advocated. In South Africa, heated hydrotherapy pools are usually available in tertiary and private healthcare settings with few or none available at primary healthcare centres and community clinics. Although there is a growing number of community pools provided for recreational purposes (Cape Town City 2018; SA Sports & Recreation 2018), these are usually not heated. No studies were found that support the use of cold aquatic therapy in the treatment of knee OA; however, the effects of water regardless of temperature have been shown to provide improvements in joint movement and flexibility because of its properties (Bender et al. 2005; Vaile et al. 2008; Walker 2011). For better outcomes, people undergoing hydrotherapy in a non-heated pool will benefit from improvements in joint movement and flexibility, but will need to combine this intervention with land-based exercises to achieve improvements in muscle strength and joint pain (Lund 2008; Wang et al. 2007).

Converse to the findings of Lund et al. (2008) and similar to this study’s results, a more recent systematic review by Waller et al. (2014) demonstrated perceivable benefit of therapeutic aquatic exercises in patients with OA of the lower limb, including the knee. The benefits described were small but significant improvements in pain, stiffness, activity levels and self-reported and objective functioning after the cessation of the therapeutic interventions with no significant improvements in muscle strength. A follow-up systematic review by Diederichs, Berger and Bartels (2010) concurred that aquatic exercises have small and mostly short-term positive effects on impairments and physical function in patients with lower limb OA. Participants in our study showed an improvement in pain and self-perceived physical function, but the duration of the effects was not determined as the participants were not followed up. There could be value in following up the participants (Lund 2008) to assess the effects of the intervention over time.

Rehabilitation of physical impairments, such as pain and stiffness, in patients with OA should be based on the principles of the International Classification of Functioning, Disability and Health (ICF) that can be translated into assessing how the patient performs or carries out activities on a daily basis in their normal environment (World Health Organization [WHO] 2010). This study did not use specific ICF principles to evaluate outcomes and the results are described according to changes in the levels or degree of impairment and activity only leaving the category of societal participation less described. The purpose of assessing and improving impairments, such as pain and stiffness, is ultimately to improve physical activity functioning to enhance participation in ADL as well as society.

The positive effects of hydrotherapy exercises demonstrated in this study may be attributed to the properties of hydrodynamic principles such as buoyancy, viscosity, turbulence and water temperature (Becker 2009). The effect of buoyancy reduces loading across painful joints and allows for better performance of functional closed-chain exercises that otherwise may be too difficult to perform on land. Buoyancy results in a sense of weightlessness promoting a perception of less stiffness in the joint and hence increasing activity (Becker 2009).

The warmth and the pressure of the water have also been reported to further assist with pain relief, decrease of swelling and ease of movement (Hinman et al. 2007). In our study, the temperature was set at 34 °C, which created a positive impact on the relaxation of muscles and joint protection, thus loosening stiff muscles. Water immersion induces an increase in methionine–encephalin plasma levels and, conversely, suppresses plasma b-endorphin, corticotropin and prolactin levels (Bender et al. 2005), thus creating muscle relaxation and reduced joint swelling.

The results of this study have confirmed previous research conclusions that support physiotherapy interventions such as hydrotherapy for decreasing pain, stiffness and improved ADL in people with OA of the knee joint (Diederichs et al. 2010; Vaile et al. 2008; Waller et al. 2014). Although most studies have recommended a longer term hydrotherapy exercise programme (Lin et al. 2004), this study showed that a short-term programme also offers significant reduction in osteoarthritic joint pain and improved self-perceived functional status. Few studies have evaluated hydrotherapy interventions on OA of the hip, knee or both. Our results of decreased pain and improved self-perceived function are similar to the results of others (Hinman et al. 2007; Nobi et al. 2012; Silva et al. 2008).

## Conclusion

This 4-week hydrotherapy intervention resulted in a decrease in measures of pain and a greater self-perceived level of physical function in people living with knee OA in this study. Participants reported having less knee pain and improved function and ability to fulfil their respective activities of daily living. Hydrotherapy in a heated pool appears to be a useful and effective intervention for the treatment of OA of the knee joint even when provided as a short-term intervention. This study should be followed up with a longitudinal randomised controlled study to determine the long term effects of an intervention like this.
